# Associations of different inflammatory factors with atherosclerosis among patients with psoriasis vulgaris

**DOI:** 10.3389/fmed.2024.1396680

**Published:** 2024-07-22

**Authors:** Nguyen Thi Kim Huong, Bui Long, Le Huu Doanh, Tran Minh Thoai, Nguyen Thi Hang, Le Khoi, Pham Thi Nu

**Affiliations:** ^1^Hanoi Medical University, Hanoi, Vietnam; ^2^Department of Dermatology, Friendship Hospital, Hanoi, Vietnam; ^3^Department of Interventional Cardiology, Friendship Hospital, Hanoi, Vietnam; ^4^National Dermatology Hospital, Hanoi, Vietnam; ^5^Department of Cardiology, Friendship Hospital, Hanoi, Vietnam

**Keywords:** TNF-α, hs-CRP, psoriasis vulgaris, atherosclerosis, IL-17A

## Abstract

**Background:**

This study aimed to measure the associations between different inflammatory factors, namely interleukin (IL)-17A, tumor necrosis factor (TNF)-α, and high-sensitivity C-reactive protein (hs-CRP), and atherosclerosis in patients with psoriasis vulgaris.

**Methods:**

A cross-sectional study was conducted at two hospitals in Hanoi, Vietnam. A total of 125 patients with psoriasis vulgaris and 50 healthy controls were recruited. Clinical characteristics and atherosclerosis were assessed. IL-17A, TNF-α, and hs-CRP levels were measured.

**Results:**

Psoriasis vulgaris patients with atherosclerosis had higher levels of hs-CRP (median = 1.22; interquartile range—IQR = 0.34–12.11) and IL-17A (median = 1.30; IQR = 0.43–4.28), but a lower level of TNF-α (median = 0.54; IQR = 0.13–3.41) compared to those without atherosclerosis (*p* < 0.05). Only LogIL-17A was positively related to atherosclerosis in psoriasis patients (Odds Ratio—OR = 2.16, 95% CI = 1.06–4.38, *p* < 0.05). After excluding systemically treated patients, LogIL-17A and Log TNF-α were associated with the likelihood of atherosclerosis (*p* < 0.05).

**Conclusion:**

This study suggests a link between elevated levels of IL-17A and TNF-α and subclinical atherosclerosis. Further investigation on a larger scale is required to establish the causality of this relationship.

## 1 Introduction

Psoriasis is a chronic inflammatory and immune-mediated condition affecting the skin and joints, often accompanied by multiple comorbidities (1, 2). Research indicates that individuals with psoriasis experience a reduced life expectancy of 4–5 years, primarily due to cardiovascular disease (CVD) (3). Furthermore, there is an elevated risk of myocardial infarction at an earlier age compared to those without the condition. This increased cardiovascular risk is thought to be linked to systemic inflammation, particularly in cases of moderate-to-severe psoriasis (4). As a result, conventional screening methods such as the Framingham risk score, which are based on traditional cardiovascular risk factors, may not effectively assess the risk of coronary heart disease in patients with psoriasis (5).

Atherosclerosis, characterized as a persistent inflammatory condition affecting the arterial system, represents a significant etiological factor contributing to CVD (6). Individuals with coexisting psoriasis and atherosclerosis pose a substantial burden on public health and healthcare systems on a global scale (7, 8). Identifying subclinical atherosclerosis may contribute to developing interventions aimed at halting the progression of the disease. Numerous investigations have examined subclinical atherosclerosis in specific vascular territories in patients with psoriasis (9, 10). Still, due to the systemic nature of atherosclerosis, a multi-territorial analysis can offer a more comprehensive understanding of the distribution and burden of atherosclerosis in these patients (11).

The precise pathways connecting psoriasis to atherosclerosis still need to be explained. However, it is evident that excessive activation of both innate and adaptive immune responses plays a significant role in the pathogenesis of both conditions (8, 12). Several studies in clinical research have demonstrated that the use of interleukin (IL)-17A inhibitors in treating psoriasis also contributes to a reduction in cardiovascular risk among patients with this condition (13, 14). Several studies have identified additional biomarkers, including high-sensitivity C-reactive protein (hs-CRP) (15) and tumor necrosis factor (TNF)-α (16), which appear to serve as critical inflammatory mediators common to both disease processes.

The natural progression of atherosclerosis typically involves an extended subclinical phase, during which the disease is often only identified at an advanced stage or following a cardiovascular event. The timely identification of subclinical atherosclerosis and the implementation of primary prevention strategies, such as effectively managing systemic inflammation, can potentially reduce the likelihood of cardiovascular disease in individuals with psoriasis. This study aimed to measure the associations between inflammatory biomarkers, IL-17A, TNF-α, hs-CRP, and atherosclerosis in patients with psoriasis.

## 2 Materials and methods

### 2.1 Study design

A cross-sectional study was conducted at the National Dermatology Hospital and Friendship Hospital in Hanoi, Vietnam, from April 2024 to December 2023, with approval from the Institutional Review Board of Hanoi Medical University (Code: 859/GCN-HDDDNCYSH-DHYHN). The study involved patients diagnosed with psoriasis vulgaris, a common type of psoriasis, who were directed to these hospitals for evaluation and treatment. To participate, patients had to be at least 18 years old, have a confirmed diagnosis of psoriasis vulgaris, complete all required clinical and laboratory evaluations, not take statin medications, and be willing to participate. The research included 125 patients who met these criteria and were selected through convenient sampling. Additionally, 50 healthy individuals aged 18 or older without health conditions were recruited as a control group.

### 2.2 Data collection method

Data regarding the diagnosis of psoriasis vulgaris in patients were obtained from their respective medical records. The clinical dataset encompasses demographic details such as age, gender, and occupation, as well as medical information including the type and duration of the illness, family and personal medical histories, smoking habits, comorbidities, body mass index, and clinical manifestations such as itching, pain, and skin redness. The degree of skin redness was categorized as mild, moderate, or severe. Additional clinical data collected included accompanying injuries (fingernails, toenails, and joints) and current treatment therapies. Laboratory examinations included cholesterol, triglycerides, LDL-c, HDL-c levels, and hs-CRP. Both research groups conducted examinations to evaluate inflammatory factors, including TNF-α and IL-17A.

Blood samples were collected from all participants to evaluate inflammatory cytokines. These samples were processed and stored under standardized conditions to ensure the integrity of the biomarkers. The following procedure was used to separate serum and measure cytokines: Blood samples were placed in an incubator at 37°C for 30 min (or at room temperature if no incubator was available), centrifuged at 5,000 rpm for 15 min, and then the serum was carefully separated from the clot using a pipette. The serum was transferred into 1.5 mL Eppendorf tubes, centrifuged at 5,000 rpm for another 15 min, and the clear serum was transferred into new Eppendorf tubes. The tubes were labeled with patient information and stored at −80°C until analysis. The levels of inflammatory cytokines, including TNF-α and IL-17A, were measured using the Luminex multiplex assay (Human Procartaplex™ Simplex Kit) according to the manufacturer’s instructions (Invitrogen™).

The severity of psoriasis vulgaris is commonly assessed using the PASI score. The PASI is a composite assessment tool that evaluates psoriasis severity in distinct anatomical regions: the head, arms, trunk, and legs. Each area is assessed for three plaque attributes: the extent of erythema (redness), induration (thickness), and desquamation (scaling). The cumulative scores of the clinical signs within each anatomical area are aggregated and adjusted based on the proportional representation of the respective area on the body. The resulting weighted scores are transformed into a final composite score, ranging from 0 to 72. The categorization of psoriasis severity is as follows: (1) Mild severity: PASI < 10; (2) Moderate severity: 10 ≤ PASI < 20; (3) Severe severity: PASI ≥20 (17).

Patients underwent arterial duplex ultrasound on the carotid, upper, and lower extremities using the Vivid E95 color Doppler ultrasound machine to diagnose arterial atherosclerosis. The diagnostic criteria for atherosclerosis included a thickening of the intima-media layer of the arteries by at least 50% compared to the surrounding intima-media layer or a thickening of the intima-media layer by more than 1.5 mm with inward bulging toward the lumen of the artery.

### 2.3 Statistical analysis

Stata version 16.0 was utilized for data analysis. Descriptive statistics were employed to calculate the mean, standard deviation, median, interquartile range (IQR), frequency, and percentage. Chi-squared, Mann–Whitney, and Kruskal-Wallis tests were used to measure differences. Univariate and multivariate logistic regression models were performed to identify associations between hs-CRP, IL-17A, TNF-α, and atherosclerosis in psoriasis vulgaris patients. Other independent variables include duration of disease, body mass index, comorbidity, plaque psoriasis, and cholesterol and triglyceride concentrations. The multivariate regression models were adjusted to age and gender. Given that the data for hs-CRP, IL-17A, and TNF-α were non-normally distributed, a logarithmic transformation was performed for these variables. A *p* value of <0.05 was considered statistically significant.

## 3 Results

Among 125 patients with psoriasis vulgaris, 48.8% had atherosclerosis, while no one had atherosclerosis in the control group. At sampling, no patients were using Secukinumab (Fraizeron). For patients with a history of using Secukinumab (Fraizeron), it was ensured that they had discontinued the medication at least 1 year before participating in the study. In reality, patients were monitored and treated monthly. All patients treated with cyclosporine and acitretin had their blood drawn early in the morning, at least 12 h after the last dose, and 7 days (168 h) for methotrexate. Patients were typically prioritized for methotrexate treatment if there was associated joint damage, acitretin if there were no prior lipid disorders, or cyclosporine if there was no history of hypertension. The average treatment duration for these methods was 1–2 years. For the group of patients using secukinumab or adalimumab, they had stopped treatment about 1 year prior (self-discontinued). They were following a treatment regimen using topical medications or combined with UV therapy.

Table 1 shows that there is no difference in age between healthy controls and psoriasis vulgaris patients. The proportion of females in the control group was significantly higher than in the patient group (*p* < 0.01). The hs-CRP concentration in the patient group was higher than in the control group (*p* < 0.05).

**Table 1:** Demographic characteristics of participants.

	Healthy controls (*n* = 50)	Psoriasis vulgaris (*n* = 125)	*p*-value
Age, year, Mean (SD)	40.2 (7.0)	43.7 (12.6)	0.06
Gender, Female, *n* (%)	39 (78.0)	46 (36.8)	<0.01
Duration of psoriasis, year, Mean (SD)	-	13.0 (9.3)	
Body mass index, kg/m^2^, Mean (SD)	-	22.4 (3.2)	
PASI score, Mean (SD)	-	9.2 (6.4)	
Having any comorbidity, *n* (%)	-	10 (8.0)	
Type of psoriasis vulgaris, Plaque, *n* (%)	-	115 (92.0)	
Cholesterol (mmol/L), Mean (SD)	-	5.3 (1.0)	
Triglyceride (mmol/L), Mean (SD)	-	2.2 (1.4)	
HDL-c (mmol/L), Mean (SD)	-	1.3 (0.5)	
LDL-c (mmol/L), Mean (SD)	-	3.1 (1.5)	
hs-CRP (pg/mL), Mean (SD)	2.1 (5.3)	2.2 (3.3)	0.04
IL-17A (pg/mL), Mean (SD)	1.5 (0.9)	1.5 (1.2)	0.42
TNF-α (pg/mL), Mean (SD)	0.9 (1.0)	1.3 (1.5)	0.16
**Treatment**			
Topical (corticosteroids, calcipotriol, or moisturizers), *n* (%)		46 (36.8)	
Secukinumab, *n* (%)		7 (5.6)	
Cyclosporine, *n* (%)		4 (3.2)	
UVA/UVB, *n* (%)		8 (6.4)	
Methotrexate, *n* (%)		39 (31.2)	
Acitretin, *n* (%)		30 (24.0)	

Table 2 reveals that the median (IQR) of hs-CRP in psoriasis vulgaris patients without and with atherosclerosis was 0.81 (0.25–6.36) and 1.22 (0.34–12.11) pg/mL, respectively (*p* < 0.05). The median hs-CRP serum level in healthy controls was significantly lower than in psoriasis vulgaris patients without atherosclerosis (*p* < 0.05) but not in those with atherosclerosis (*p* > 0.05). The median (IQR) of IL-17A in psoriasis vulgaris patients without and with atherosclerosis was 1.19 (0.43–2.66) and 1.30 (0.43–4.28) pg/mL, respectively (*p* < 0.05). The median IL-17A serum level in healthy controls was significantly lower than in psoriasis vulgaris patients with atherosclerosis (*p* < 0.05) but not in those without atherosclerosis (*p* > 0.05). The median (IQR) of TNF-α in psoriasis vulgaris patients without and with atherosclerosis was 1.10 (0.13–4.60) and 0.54 (0.13–3.41) pg/mL, respectively (*p* < 0.05). The median TNF-α level in healthy controls was significantly lower than in psoriasis vulgaris patients without atherosclerosis (*p* < 0.05) but not in those with atherosclerosis (*p* > 0.05).

**Table 2:** hs-CRP, IL-17A, and TNF-α in healthy controls, psoriasis patients with and without atherosclerosis.

Biomarkers		Healthy control (*n* = 50)	Psoriasis without atherosclerosis (*n* = 64)	Psoriasis with atherosclerosis (*n* = 61)
hs-CRP (pg/mL)	Mean (SD)	2.06 (5.30)	1.64 (2.06)	2.84 (4.16)
	Median	0.74	0.81	1.22
	IQR (25–75th)	0.31–3.54	0.25–6.36	0.34–12.11
	*p*	*p*(1) = 0.22; *p*(2) < 0.01; *p*(3) = 0.01		
IL-17A (pg/mL)	Mean (SD)	1.51 (0.86)	1.41 (1.32)	1.66 (1.00)
	Median	1.30	1.19	1.30
	IQR (25–75th)	0.43–2.66	0.43–2.66	0.43–4.28
	*p*	*p*(1) = 0.02; *p*(2) = 0.27; *p*(3) < 0.01		
TNF-α (pg/mL)	Mean (SD)	0.94 (1.00)	1.52 (1.52)	1.15 (1.48)
	Median	0.27	1.10	0.54
	IQR (25–75th)	0.13–2.25	0.13–4.60	0.13–3.41
	*p*	*p*(1) = 0.01; *p*(2) = 0.43; *p*(3) = 0.02		

*p*(1): compare between healthy control and psoriasis without atherosclerosis; *p*(2): compare between healthy control and psorisis with atherosclerosis; and *p*(3): compare between psoriasis with and without atherosclerosis.

Table 3 shows that only a significant difference in TNF-α was found between mild-psoriasis and moderate–severe-psoriasis patients with atherosclerosis (*p* < 0.05).

**Table 3:** hs-CRP, IL-17, and TNF-α in psoriasis patients with and without atherosclerosis across different levels of psoriasis severity.

Biomarkers		Without atherosclerosis			With atherosclerosis		
		Mild (*n* = 38)	Moderate–Severe (*n* = 26)	*p*- value	Mild (*n* = 37)	Moderate–Severe (*n* = 24)	*p*-value
hs-CRP (pg/mL)	Mean (SD)	1.42 (1.64)	1.96 (2.54)	0.24	2.41 (4.22)	3.50 (4.06)	0.18
	Median	0.76	1.06		1.08	1.73	
	IQR (25–75th)	0.28–3.23	0.39–4.11		0.38–4.76	0.43–7.59	
IL-17A (pg/mL)	Mean (SD)	1.55 (1.57)	1.21 (0.84)	0.09	1.54 (0.86)	1.86 (1.18)	0.25
	Median	1.30	0.86		1.30	1.30	
	IQR (25–75th)	0.43–2.66	0.43–1.79		0.86–2.66	0.86–2.78	
TNF-α (pg/mL)	Mean (SD)	1.34 (1.41)	1.77 (1.64)	0.17	0.87 (1.07)	1.58 (1.87)	0.03
	Median	1.10	1.10		0.26	1.07	
	IQR (25–75th)	0.13–3.41	0.27–3.42		0.13–3.07	0.13–3.41	

In univariate analysis, after adjusting to age and gender, Table 4 shows that patients with a higher duration of disease were more likely to have atherosclerosis (OR = 1.07, 95%CI = 1.02–1.13) logarithmic transformation of hs-CRP and IL-17A were found to be positively associated with atherosclerosis. However, after adjustment, only LogIL-17A was positively related to atherosclerosis in psoriasis vulgaris patients (OR = 2.16, 95%CI = 1.06–4.38, *p* < 0.05). No association was found between body mass index, PASI score, comorbidity, plaque psoriasis, triglycerides, or cholesterol with atherosclerosis (*p* > 0.05). After excluding systemically treated patients, LogIL-17A and Log TNF-α were associated with the likelihood of atherosclerosis (*p* < 0.05).

**Table 4:** Univariate and multivariate logistic regression to identify associations between hs-CRP, IL-17, TNF-α, and atherosclerosis in psoriasis patients.

	Univariate regression (*n* = 125)			Multivariate regression^*¶^ (*n* = 125)			Multivariate regression after excluding systemically treated patients^*¶^ (*n* = 45)		
	OR	95%CI	*p*-value	Adjusted OR	95%CI	*p*-value	Adjusted OR	95%CI	*p*-value
Duration of disease (per year)	1.08	1.03–1.12	<0.01	1.07	1.02–1.13	<0.01	1.18	1.03–1.36	0.02
Body mass index (per kg/m^2^)	1.04	0.93–1.17	0.46	1.02	0.89–1.17	0.80	1.15	0.79–1.67	0.46
PASI score	1.02	0.96–1.07	0.55	0.97	0.91–1.04	0.46	1.04	0.86–1.26	0.71
Having any comorbidity (Yes vs. No-ref)	4.68	0.95–23.00	0.06	0.54	0.82–35.30	0.08	-^**¶**^		
Plaque psoriasis (Yes vs. No.)	4.21	0.86–20.71	0.08	3.12	0.54–17.96	0.20	-^**¶**^		
Triglycerid (per mmol/L)	1.13	0.88–1.45	0.36	1.03	0.76–1.41	0.84	0.71	0.29–1.76	0.46
Cholesterol (per mmol/L)	1.31	0.90–1.92	0.16	1.55	0.98–2.44	0.06	1.80	0.69–4.72	0.23
Log hs-CRP	1.49	1.05–2.12	0.03	1.32	0.95–2.05	0.22	1.23	0.40–3.82	0.72
Log IL-17A	2.05	1.10–3.82	0.03	2.16	1.06–4.38	0.03	18.58	1.37–252.50	0.028
Log TNF-α	0.76	0.57–1.01	0.06	0.76	0.55–1.05	0.10	0.48	0.23–0.98	0.04
				*R*^2^ = 0.18			*R* = 0.42		

^*^The model was adjusted to age and gender; ^¶^No observation.

## 4 Discussion

Specific research suggests that the higher risk of cardiovascular disease in individuals with psoriasis may be due to the burden of inflammatory disease, which IL-17A influences (13, 14, 18). IL-17A contributes to the impairment of blood vessel function, and higher levels of IL-17A have been found in patients with acute coronary artery syndromes compared to those with stable coronary artery disease (19). Modern research indicates that psoriasis and atherosclerosis may share similar immune pathways related to the IL-17A axis (20). Some studies also suggest that IL-17A contributes to the reinforcement of atherosclerotic plaques. It is thought that IL-17A helps keep plaques stable by decreasing the expression of VCAM-1 on endothelial cells and preventing T-cell entry into plaques, thus reducing the secretion of inflammatory cytokines like IFN-γ and increasing levels of anti-inflammatory cytokines such as IL-5, IL-10, and TGF-β (20). The impact of IL-17A on atherosclerosis can be attributed to the inflammatory microenvironment, including Treg cells and cytokines, which determine whether it promotes plaque instability or stability. This study confirmed that IL-17A primarily had a pro-inflammatory function in the concurrent presence of psoriasis and atherosclerosis.

The present study also assessed the correlation between hs-CRP and atherosclerosis; however, no statistically significant association was observed in the multivariate analysis. Meanwhile, TNF-α was found to be associated with atherosclerosis. The presence of IFN-γ and TNF-α may augment the potential effects of IL-17A on the pathogenesis of psoriasis and atherosclerosis. The correlation between psoriasis and atherosclerosis has been conceptualized as “one syndrome and two plaques” (21) based on the analogous inflammatory cytokine profiles shared by both conditions, such as TNF-α and IL-17A (22). Furthermore, a synergistic impact of IFN-γ and TNF-α on the immunopathogenic mechanism has been observed in the concurrent presence of atherosclerosis and psoriasis (16). Meanwhile, the polypeptide hs-CRP serves as an acute-phase reactant and is primarily synthesized by hepatocytes during periods of inflammation in response to specific cytokines, including interleukin IL-1, IL-6, and TNF-α (23, 24). The high-sensitivity C-reactive protein (hs-CRP) assay demonstrates greater sensitivity in detecting small fluctuations in C-reactive protein (CRP) levels during episodes of inflammation compared to standard CRP testing, as indicated by previous research (25). Lachine and colleagues initially proposed hs-CRP as a potential indicator of subclinical atherosclerosis in individuals with type 2 diabetes mellitus (26). Furthermore, research undertaken by Su et al. affirmed a notable association between high-sensitivity C-reactive protein (hs-CRP) and subclinical carotid atherosclerosis in prediabetic adults in contrast to those with euglycemia (27). Niknezhad et al. (15) demonstrated that individuals with psoriasis are at an elevated risk for subclinical atherosclerosis and suggested that high-sensitivity C-reactive protein (hs-CRP) may serve as a valuable marker for assessing the future risk of cardiovascular diseases in this patient population. The present study observed a statistically significant elevation in serum hs-CRP levels among psoriasis patients relative to healthy controls, consistent with findings from a previously conducted investigation (15). Our study did not identify a significant association between hs-CRP and atherosclerosis, a finding that contrasts with previous research (15, 28, 29). The absence of a discovered association may be attributed to the homogeneity of patient characteristics between the two groups with and without atherosclerosis. A subsequent study with a larger sample size and diverse sample characteristics is essential to confirm the relationship between hs-CRP, TNF-α, and atherosclerosis in psoriasis patients.

In our study, only hs-CRP serum levels in psoriasis patients were significantly higher than in healthy controls. At the same time, we found no differences between both groups regarding TNF-α and IL-17A. These findings contradict other prior studies (9, 16, 22, 25). This discrepancy could be because the mean PASI score of patients was 9.2, indicating that most patients had stable disease conditions. Meanwhile, in other studies, patients were primarily selected with PASI >10, which might result in higher serum concentrations of inflammatory factors compared to our study. Moreover, the patients involved in this study predominantly utilized immunosuppressive drugs such as Methotrexate, Ciclosporin, and acitretin. Only slightly more than 30% of patients used topical medication (e.g., corticosteroids, calcipotriol, and moisturizers). This leads to lower levels of inflammatory factors compared to studies conducted internationally. In addition, a possible explanation for why IL-17A correlates with atherosclerosis only in psoriasis patients, despite similar levels in healthy individuals, could involve the chronic inflammatory environment characteristic of psoriasis (23, 24). In psoriasis patients, IL-17A may synergize with other inflammatory cytokines like TNF-α and IFN-γ, enhancing its effects on atherosclerosis (21). Additionally, target cells in the vascular system of psoriasis patients might be more sensitive to IL-17A due to disease-specific changes, such as altered gene and protein expression (30). Genetic and biological factors unique to psoriasis could further amplify IL-17A’s impact on atherosclerosis (30). These factors are not present in healthy individuals, explaining the lack of correlation in the latter group.

The primary limitation of the study is attributed to the small sample size. Utilizing a cross-sectional study design constrains the ability to assess proinflammatory cytokines and biomarkers longitudinally. Caution should be exercised when interpreting the results of the multivariate analysis due to the small sample size. Additional longitudinal research studies with a more expansive sample size and a broader array of inflammatory biomarkers and cytokines are recommended. Moreover, we did not measure the relationship between the development of atherosclerosis and other variables such as BMI, comorbidities, or cholesterol and triglyceride values, nor did we measure the relationship between inflammatory cytokine expression and these same variables. Further studies should be warranted to address these knowledge gaps.

## 5 Conclusion

The findings of our study suggest a possible link between elevated levels of IL-17A and subclinical atherosclerosis. However, further investigation on a larger scale is required to establish the causality of this relationship. The role of systemic inflammation is significant in the development of both psoriasis and atherosclerosis. Anti-inflammatory therapies not only hold a pivotal position in the management of psoriasis but also can mitigate the risk of cardiovascular diseases by decreasing inflammatory markers such as IL-17A.

## Data availability statement

The raw data supporting the conclusions of this article will be made available by the authors, without undue reservation.

## Ethics statement

The studies involving humans were approved by Institutional Review Board of Hanoi Medical University (Code: 859/GCN-HDDDNCYSH-DHYHN). The studies were conducted in accordance with the local legislation and institutional requirements. The participants provided their written informed consent to participate in this study.

## Author contributions

NHu: Conceptualization, Formal analysis, Investigation, Methodology, Writing – original draft, Writing – review & editing. BL: Conceptualization, Methodology, Supervision, Validation, Writing – original draft, Writing – review & editing. LD: Conceptualization, Supervision, Validation, Writing – original draft, Writing – review & editing. TT: Formal analysis, Investigation, Methodology, Validation, Writing – original draft, Writing – review & editing. NHa: Investigation, Project administration, Resources, Visualization, Writing – original draft, Writing – review & editing. LK: Investigation, Methodology, Project administration, Visualization, Writing – original draft, Writing – review & editing. PN: Data curation, Investigation, Project administration, Writing – original draft, Writing – review & editing.
